# Porphyrin Cobalt(III) “Nitrene Radical” Reactivity; Hydrogen Atom Transfer from *Ortho*-YH Substituents to the Nitrene Moiety of Cobalt-Bound Aryl Nitrene Intermediates (Y = O, NH)

**DOI:** 10.3390/molecules21020242

**Published:** 2016-02-20

**Authors:** Monalisa Goswami, Christophe Rebreyend, Bas de Bruin

**Affiliations:** Van ‘t Hoff Institute for Molecular Sciences, University of Amsterdam, Science Park 904, 1098 XH Amsterdam, The Netherlands; m.goswami@uva.nl (M.G.); C.Rebreyend@uva.nl (C.R.)

**Keywords:** aryl azides, cobalt(II) porphyrins, nitrene radicals, hydrogen atom transfer, azabenzenes, *ortho*-iminoquinonoid, phenylene diimine

## Abstract

In the field of cobalt(II) porphyrin-catalyzed metallo-radical reactions, organic azides have emerged as successful nitrene transfer reagents. In the pursuit of employing *ortho*-YH substituted (Y = O, NH) aryl azides in Co(II) porphyrin-catalyzed nitrene transfer reactions, unexpected hydrogen atom transfer (HAT) from the OH or NH_2_ group in the *ortho*-position to the nitrene moiety of the key radical-intermediate was observed. This leads to formation of reactive *ortho*-iminoquinonoid (Y = O) and phenylene diimine (Y = NH) species. These intermediates convert to subsequent products in non-catalyzed reactions, as is typical for these free organic compounds. As such, the observed reactions prevent the anticipated cobalt-mediated catalytic radical-type coupling of the nitrene radical intermediates to alkynes or alkenes. Nonetheless, the observed reactions provide valuable insights into the reactivity of transition metal nitrene-radical intermediates, and give access to *ortho*-iminoquinonoid and phenylene diimine intermediates from *ortho*-YH substituted aryl azides in a catalytic manner. The latter can be employed as intermediates in one-pot catalytic transformations. From the *ortho*-hydroxy aryl azide substrates both phenoxizinones and benzoxazines could be synthesized in high yields. From the *ortho*-amino aryl azide substrates azabenzene compounds were obtained as the main products. Computational studies support these observations, and reveal that HAT from the neighboring OH and NH_2_ moiety to the nitrene radical moiety has a low energy barrier.

## 1. Introduction

In the context of homogeneously catalyzed organic transformations carbene and nitrene transfer reactions provide interesting possibilities [1,2,3,4,5,6,7]. In these transformations metal catalysts that can activate carbene or nitrene precursors are employed, typically leading to the formation of metal *carbenoid* or *nitrenoid* species. The thus formed *carbenoids* and *nitrenoids* can then add to a variety of unsaturated bonds in alkenes and alkynes. In addition, metal-catalyzed carbene transfer can be used in reaction in which carbene moieties formally insert into X−H bonds (X = O, N, S, Si) [2]. Given the ubiquity of nitrogen atoms in biologically active compounds, it is also interesting to find ways in which C–N bonds can be formed by addition or insertion of nitrenoids to a C=C or C≡C bond or into a C–H bond. Metal-catalyzed nitrene transfer reactions are therefore promising and worth investigating.

Cobalt(II) porpyhrins have proven to be quite successful in this field [2,8,9,10,11,12,13,14,15,16,17,18,19,20,21,22,23,24,25]. Besides being efficient in carbene transfer reactions, cobalt(II) porphyrins have also attracted attention in several nitrene transfer reactions [11,14,15,16,18,21,22,26,27,28]. In the early stages of this type of catalysis it was quite common to use iminoiodanes or haloamine-T compounds as nitrene sources for transfer to other molecules [29,30,31,32,33]. These are, however, not the most convenient and benign nitrene sources, as they are poorly soluble and lead to the formation of undesirable side products like phenyl iodide and other halogen-containing compounds. Organic azides are potentially more interesting nitrene-transfer reagents [3,5,6], as they are more soluble and the only byproduct formed during the generation of nitrenes from azides is dinitrogen.

**Scheme 1:**
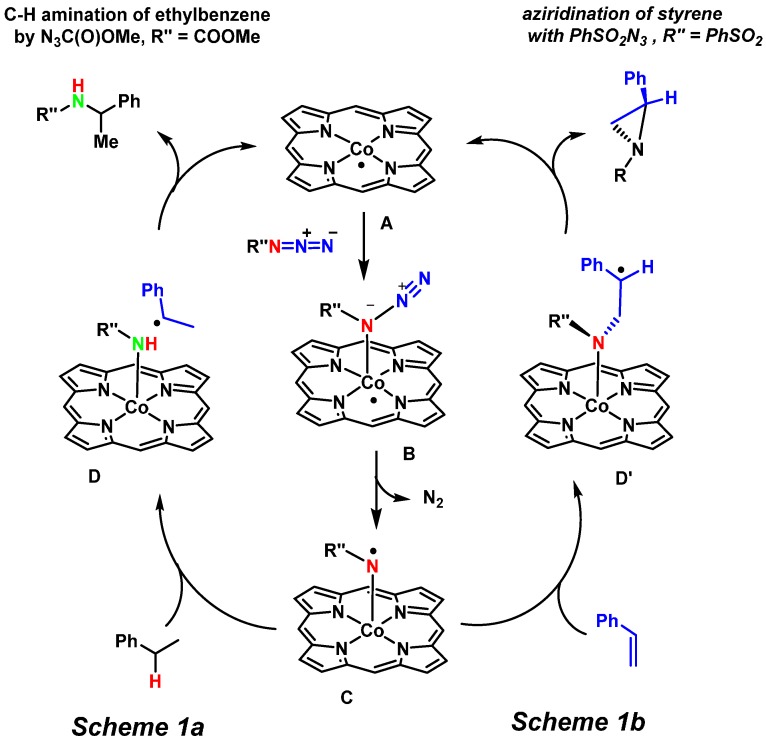
Mechanisms of cobalt(II) porphyrin catalyzed amination (**a**) and aziridination (**b**).

Mechanistically these systems are quite unique as they operate via cobalt(III) nitrene radical species, formed by electron transfer from cobalt(II) to the nitrene moiety upon activation of the azide. This leads to discrete radical character of the reactive nitrenoid intermediate. The detailed mechanistic aspects of two such catalytic nitrene transfer reactions with cobalt porphyrins have been elucidated by our group previously (Scheme 1) [12,18,34,35]. The mechanism of C−H bond amination of ethylbenzene, toluene, and tetralin (1,2,3,4- tetrahydronaphthalene) was studied with a variety of organic azides like N_3_C(O)OMe, N_3_SO_2_Ph, *etc.* using density functional theory (DFT) and electron paramagnetic resonance (EPR) spectroscopy (Scheme 1a) [18]. The mechanism of cobalt(II) porphyrin-mediated aziridination of styrene with PhSO_2_N_3_ was also studied (Scheme 1b) [21]. For both amination and aziridination reactions, the DFT calculations revealed a stepwise radical process involving coordination of the azide to the cobalt(II) center, followed by release of dinitrogen to produce an unusual “nitrene radical” intermediate **C** (Scheme 1). In addition, experimental EPR spectroscopic studies, combined with DFT EPR property calculations being in good agreement with the experimental data, revealed the formation of a (por)Co^III^−N**•**Y nitrene radical adduct **C** from the catalyst in the presence of an excess of the azide in benzene. Formation of a nitrene moiety at cobalt(II)porphyrin effectively leads to electron transfer from the metal to the nitrene, thus reflecting the redox non-innocence of the nitrene substrate ligand. The spin density of this intermediate resides almost entirely on the nitrogen atom of the nitrene moiety. More recently, the intermediate **C** was fully characterized using an array of spectroscopic methods [12].

**Scheme 2:**
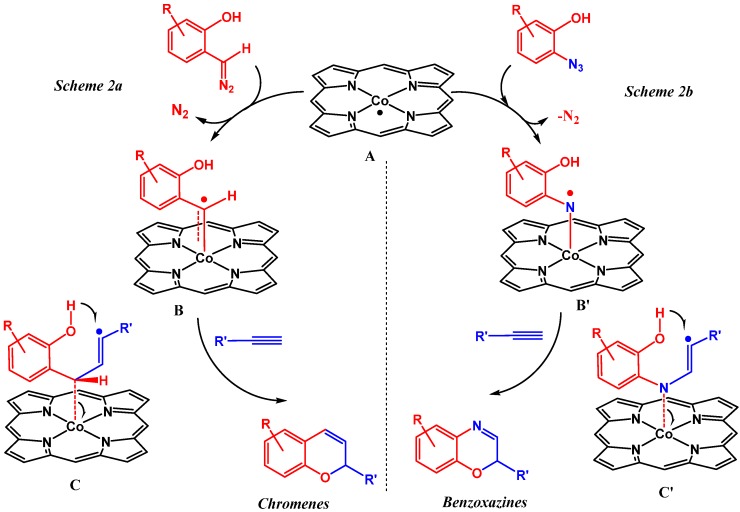
Proposed mechanism of previously reported catalytic 2*H*-chromene formation based on DFT calculations (**a**); and the envisioned related pathway for the synthesis of benzoxazines using ortho-hydroxy-aryl azides (**b**).

In order to expand the scope of cobalt(II) porphyrin catalyzed nitrene transfer reactions we sought to use *ortho-*substituted aryl azides as substrates, which on activation by Co(II) porphyrins could undergo subsequent reactions with unsaturated bonds. The idea was in part inspired by our work on related *ortho-*hydroxyaryl carbene radicals [20]. Activation of the carbene precursor and subsequent reaction of the thus generated cobalt(III) carbene radical with phenyl acetylene allowed us to synthesize 2*H*-chromenes in good yields (Scheme 2a). The mechanism of this reaction is particularly interesting, and DFT calculations reveal the formation of the salicyl−vinyl radical intermediate by a metalloradical-mediated process. Unexpectedly, subsequent hydrogen atom transfer (HAT) from the hydroxyl moiety to the vinyl radical leads to formation of an *o*-quinone methide intermediate. This intermediate dissociates immediately from the metal center. This then undergoes an endocyclic, sigmatropic ring-closing to give the chromene (Scheme 2a). This was confirmed by EPR experiments and radical poisoning experiments using the 2,2,6,6-tetramethyl-1-piperidinyloxylyl (TEMPO) free radical. This reaction inspired us to extend the protocol to related ring-closing reactions, but this time using a nitrene precursor instead of a carbene precursor. This was expected to produce heterocycles like benzoxazines, which find applications in the pharmaceutical industry (Scheme 2b). The reactions, however, take a different course than expected and these unexpected results of the investigations are disclosed in this paper.

## 2. Results and Discussion

### 2.1. Experimental Studies

**Figure 1:**
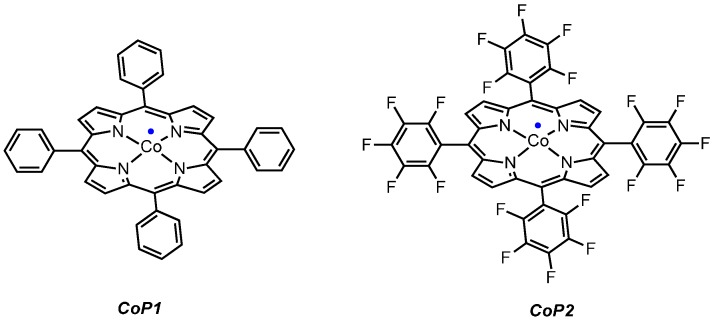
Different cobalt(II) porphyrins employed in this study.

**Scheme 3:**
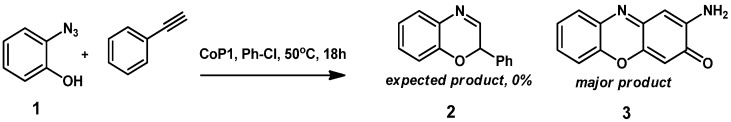
Formation of phenoxizinone **3** from *o*-azidophenol **1** and **CoP1**.

As an initial test reaction, *o*-azidophenol **1** was reacted with **CoP1** (Figure 1) in the presence of phenyl acetylene, expecting formation of benzoxazine **2** as the product (Scheme 3). However, on analyzing the crude reaction mixture the expected product **2** was not detected. Instead, new ^1^H-NMR peaks were detected, and the azide was fully consumed. The main product in the reaction was separated from the reaction mixture by column chromatography, and proved to be phenoxizinone **3** (Scheme 3).

**Scheme 4:**
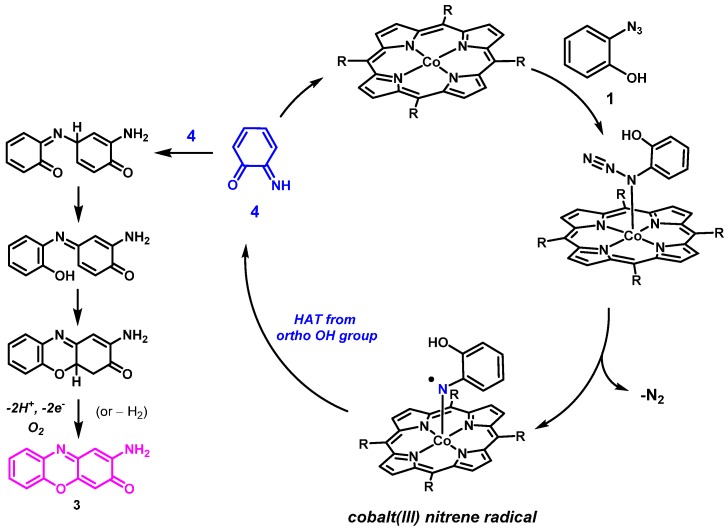
Proposed mechanism for formation of **3** from **1** mediated by **CoP1**.

The unexpected formation of product **3** can be rationalized by considering HAT from the hydroxyl group in the *ortho*-position of the cobalt(III) nitrenoid intermediate formed on activation of the azide by the catalyst **CoP1**. This should result in the formation of the reactive *ortho*-imino-quinonoid compound **4** (Scheme 4). Attack of the imine N atom of **4** on the para position (w.r.t to the carbonyl group) of another molecule of **4**, followed by 1,5-sigmatropic migration of a H atom, subsequent deprotonation and oxidation would lead to formation of the phenoxazine product 3. Such a pathway using *ortho*-aminophenol (OAP) has been reported before using manganese porphyrins that use H_2_O_2_ as the external oxidant [36]. In our case, the deprotonation and oxidation steps probably took place in air during column chromatography.

Compound **3** has anti-inflammatory and immunomodulatory properties and is, therefore, valuable for its medicinal properties. As mentioned before, other reported methods involving metalloporphyrin-catalyzed synthesis of **3** from OAP make use of more powerful oxidants like hydrogen peroxide, and are believed to be formed via different mechanisms [36].

To see if formation of **3** could be avoided by using an excess of the alkyne, the reaction was also performed in neat alkyne. However, also in this case almost exclusive formation of compound **3** was observed. In addition to that, simple aziridination of alkenes like cyclohexene and styrene were also attempted using *o-*azidophenol **1**, which in all cases led to formation of only one identifiable and major product, the phenoxizinone **3** (Table 1).

**Scheme 5:**
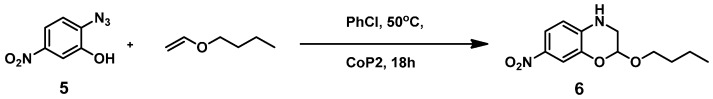
Trapping of the *o*-quinone monoimine in an IEDDA reaction.

To be able to trap the proposed reactive intermediate **4** with a different substrate we decided to protect the phenyl ring on the 5-position, so that coupling of two iminoquinones to give products like **3** is prevented. As such, 2-azido-5-nitrophenol **5** was synthesized. As a test reaction we tried to trap the corresponding *o*-quinone monoamine intermediate with 1-butoxyethene in an Inverse Electron Demand Diels Alder (IEDDA) reaction (Scheme 5). For this transformation, however, no fruitful results were obtained when using **CoP1** as the catalyst. The crude reaction mixtures showed formation of a mixture of products containing a lot of azide starting material. This is most likely due to the inherent inability of **CoP1** to activate the azide under the applied reaction conditions. However, on switching to **CoP2**, the desired transformation could be achieved and product **6** was obtained in 80% yield. Non-catalytic trapping of *o*-quinone monoimines has been reported earlier, but the method uses stoichiometric amounts of halogen containing oxidants, which is avoided in the reaction depicted in Scheme 5 [37].

Substrate **5** was also tested in reactions with other potential reaction partners, in which we expected to be able to trap the iminoquinone intermediate with different C=C and C≡C bonds (Table 2). With phenyl acetylene no reaction involving the C≡C bond was observed. Other alkenes also proved unreactive in this process. Apparently, only electron rich alkenes like 1-butoxyethene are suitable reaction partners in this process.

**Scheme 6:**
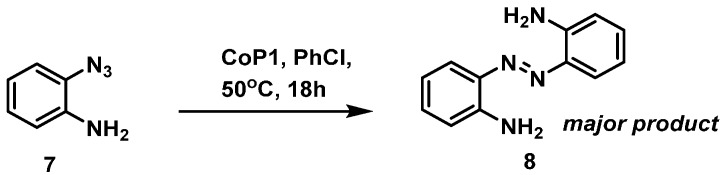
Reaction of o-aminophenyl azide **7** with cobalt(II) porphyrin catalyst **CoP1**.

To investigate the generality of the observed HAT reactivity, we decided to explore the reactivity of aryl azide **7**, containing an NH_2_ substituent in the *ortho*-position instead of an OH substituent. The *o*-amino phenylazide **7** was synthesized and tested under the same reaction conditions, using **CoP1** as the catalyst (Scheme 6). In contrast to our expectations, however, azabenzene **8** was obtained as the major product. This observation intrigued us, as formation of aza compounds as products in reaction of azides with cobalt(II) porphyrin catalyzed processes has been reported only once before, and only as a minor side product [38].

**Scheme 7:**
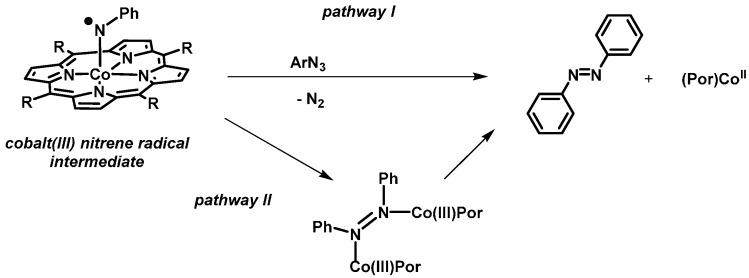
Proposed mechanisms for formation of aza compounds in cobalt(II) porphyrin catalyzed reactions with organic azides.

Two mechanistic proposals for formation of aza compounds in cobalt(II) porphyrins catalyzed reactions have been suggested: (I) Attack of the azide starting compound on the nitrene intermediate, producing azabenzene with simultaneous N_2_-loss, and (II) dinuclear N-N coupling involving two nitrene complexes (Scheme 7).

It is not so clear which reaction conditions lead to the formation of azabenzenes and which not. One suggested possibility was that in the presence of a large excess of another reaction partner, the concentration of the nitrene intermediate cannot build-up in a sufficient manner to allow formation of aza compounds via dinuclear N–N coupling [39]. To check this hypothesis, we repeated the reaction of azide **7** in the presence of a large excess of phenyl acetylene or styrene in refluxing toluene. However, once again the aza compound **8** was obtained as the major product. This led us to believe that there is something unique about the NH_2_ substituent in azide **7** that relates to formation of only the aza compound. We speculate that this relates to rapid formation of *o-*phenylenediimine (OPDI) undergoing further reactions to form **8**.

**Scheme 8:**
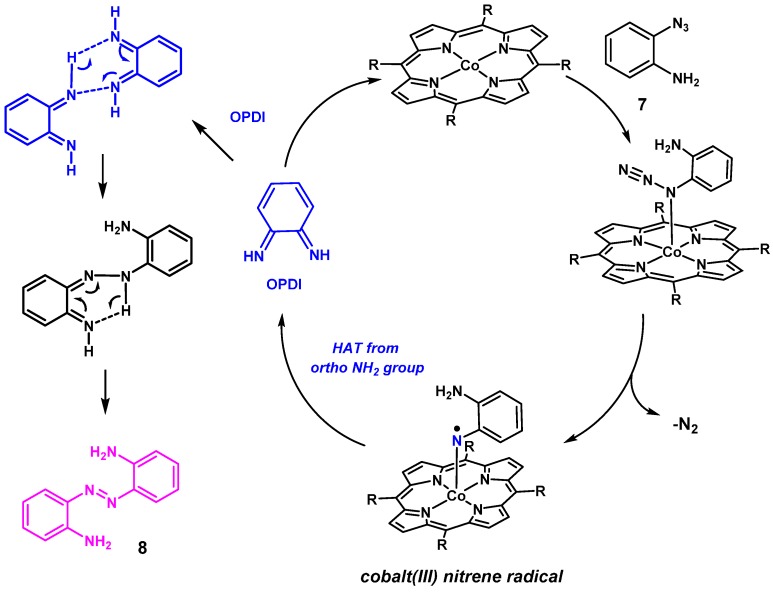
Proposed mechanism of formation of azabenzene **8** from azide **7** involving the intermediate OPDI.

The formation of azabenzene **8** from *ortho*-aminophenyl azides via the proposed phenylene diimine intermediate **9** can be reasoned in the mechanism depicted in Scheme 8. Activation of azide **7** by the cobalt(II) catalysts leads to formation of a reactive nitrene radical intermediate. Subsequent rapid HAT from the ortho-NH_2_ group to the nitrene radical moiety in this intermediate produces OPDI. This is followed by intermolecular coupling of two OPDI molecules to form aza compound **8**. The ODPI coupling steps could also be catalyzed by trace amounts of orthophenylenediamine (generated by some minor side-reaction), as has been suggested in literature [40].

It is worth mentioning that this reaction of *ortho*-amino substituted phenyl azides to give the azabenzene compounds as the major product is one of the few catalytic examples reported so far to synthesize aza compounds in high yields [41,42,43]. An example involving a ruthenium metallo-radical system proceeds via a free nitrene intermediate and works catalytically only for aryl azides with electron rich substitutents like OMe and OEt [43]. Another iron based example is limited in the sense that only azides with bulky substitutents like mestiyl groups result in formation of aza compounds [41]. With trifluoromethyl and methyl substituents dimers of the metal complex are obtained. The other example involves a nickel complex, but this system produces only stoichiometric amounts of aza compounds [42].

### 2.2. Computational Studies

To estimate the reaction barriers for the assumed facile HAT process from the *ortho*-YH substituent (Y = O, NH) to the nitrene moiety, we investigated this process with DFT for both the OH and the NH_2_ substituents.

**Figure 2:**
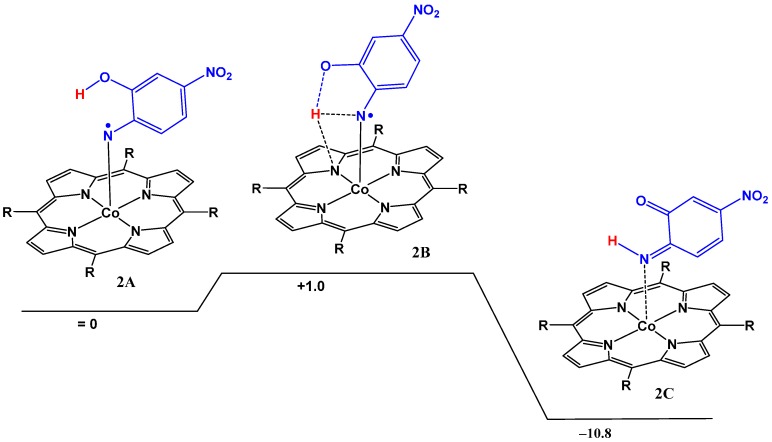
The DFT calculated barrier for HAT from the ortho hydroxyl group to the nitrene moiety. ΔG°_298K_ in kcal·mol^−1^, calculated at the BP86, def2-TZVP level with dispersion corrections.

Starting from the cobalt(III) nitrene radical species the intramolecular HAT reaction of the cobalt(III)nitrene radical of the azide **5** was found to proceed via a 6-membered transition state, further stabilized by a hydrogen bonding interaction between the transferred hydrogen atom and a pyrrole nitrogen atom of the porphyrin (Figure 2, Computational details in Table S1). The barrier is very low (+1.0 kcal·mol^−1^), thus explaining the experimental observations. Overall, the HAT process is exergonic by −10.8 kcal·mol^−1^.

**Figure 3:**
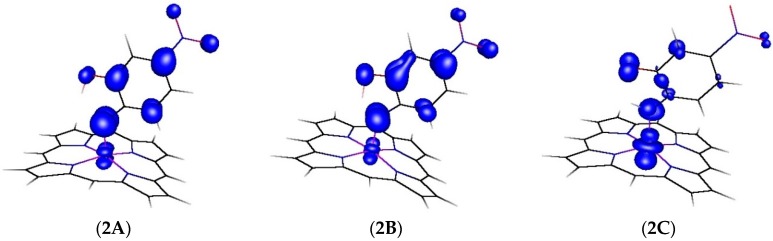
Changes in the spin density distribution during the HAT process shown in Figure 2.

We further evaluated the changes in spin density distribution (see Figure 3 and Table 3) during the HAT process (see also Figure 2). The spin density distribution of the transition state is very similar to that in the initial cobalt(III) nitrene radical, but after the HAT barrier and transfer of the hydrogen atom from the –OH group, most of the spin density moves back to cobalt (Figure 3 and Table 3). Simultaneously, the bond length of the Co-N bond elongates from 1.818 Å in the nitrene radical to 1.923 Å in the imide as depicted in Table 4.

**Figure 4:**
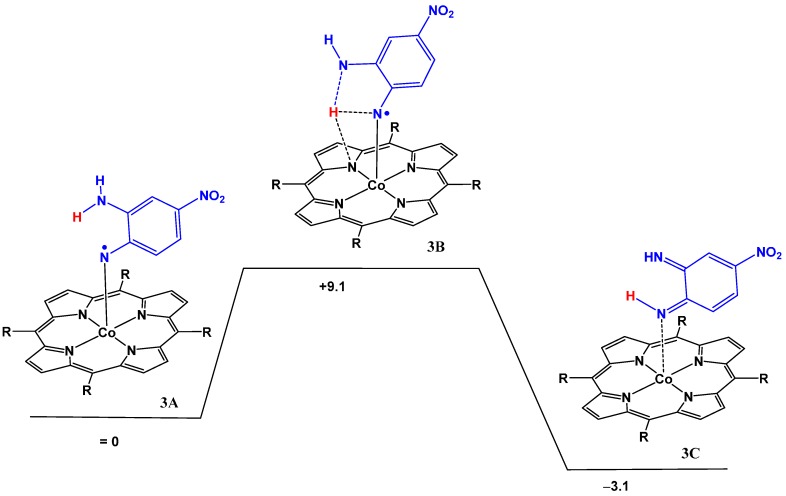
The DFT calculated barrier for HAT from the *ortho*-amino group to the nitrene moiety. ΔG°_298K_ in kcal·mol^−1^, calculated at the BP86, def2-TZVP level with dispersion corrections.

The almost barrierless abstraction of a neighboring hydrogen atom by the reactive cobalt(III) *nitrene radical* thus prevents any intermolecular coupling reactions of the nitrene moiety with exogenous substrates. A similar process was also calculated for the NH_2_ substituted azide, and once again the barrier for intramolecular HAT between the NH_2_ group and the nitrene moiety was found to be low (see Figure 4). The free energy required to reach the transition state is only +9.1 kcal·mol^−1^ implying that this intramolecular process is fast, even at room temperature. The overall transformation is exergonic by −3.1 kcal·mol^−1^.

**Figure 5:**
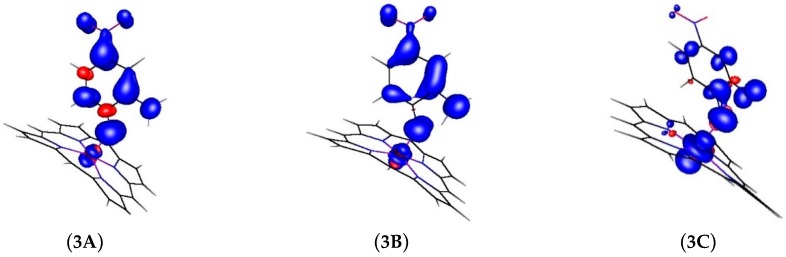
Changes in spin density distribution for HAT depicted in Figure 4.

The computed changes in the spin density distributions are once again in line with the HAT process, and similar to those computed for HAT from the OH substituent (See Figure 5 and Table 5). During the HAT process the spin population shifts from the nitrene radical in **3A** to cobalt in **3C**, and after the HAT process the spin density is mostly concentrated at the cobalt atom. The bond distance analysis of the relevant bonds are shown in Table 6. Here the Co-N bond in the final structure elongates from 1.8521 Å in the transition state **3B** to 1.9604 Å in the final structure **3C**. Interestingly, the adduct remains coordinated to the cobalt complex, as is evident from the bond distances.

**Figure 6:**
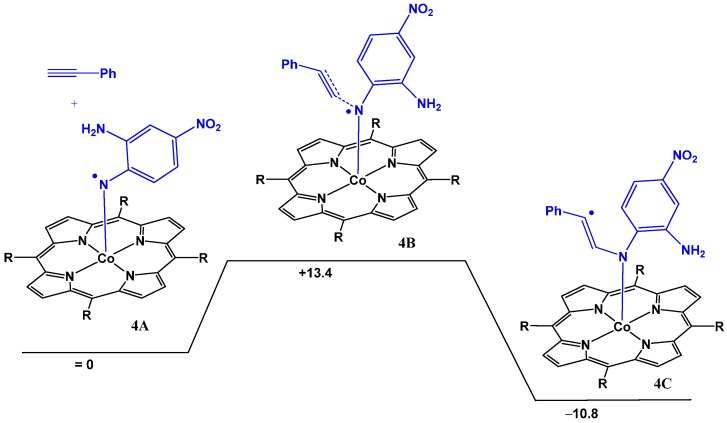
DFT computed reaction barrier for attack of the cobalt(III) nitrene radical on phenyl acetylene leading to formation of a γ-alkyl radical intermediate. ΔG°_298K_ in kcal·mol^−1^ computed at the BP86, def2-TZVP level with dispersion corrections.

While the barrier for the HAT process depicted in Figure 4 is low, it is not barrierless. Hence, to exclude the possibility of intermolecular coupling of the nitrene radical to phenyl acetylene (see Scheme 2b) being in competition with the intramolecular HAT process (Figure 4), we decided to make a direct computational comparison of the two processes. Attack of the porphyrin cobalt(III) nitrene radical on the alkyne to form the γ-radical species **4C** (**C’** in Scheme 2b) was computed at the same DFT level (see Figure 6). The latter process is exergonic (−10.8 kcal·mol^−1^), but has a computed barrier of +13.4 kcal·mol^−1^. This barrier is almost 4 kcal·mol^−1^ higher than the barrier for intramolecular HAT (see Figure 4), and hence this process cannot efficiently compete with the intramolecular HAT reaction. Formation of the OPDI intermediate by HAT is expected under all reaction conditions.

**Figure 7:**
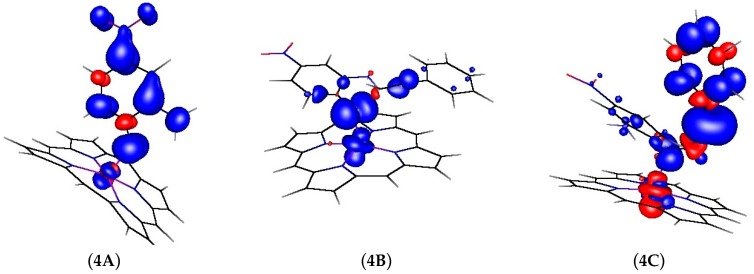
Changes in spin density distributions during the HAT depicted in Figure 6.

The spin density distribution changes were once again calculated for the structures **4A**, **4B** and **4C**. Interestingly, in contrast to the structures shown in Figure 3 and Figure 5, the spin density in structure **4C** is strongly delocalized over the carbon atoms of the γ-alkyl radical and the adjacent phenyl ring, with barely any spin density at the cobalt atom (see Figure 7).

## 3. Experimental Section

### 3.1. General Information

All chemicals were purchased from commercially available sources unless otherwise mentioned. Solvents for reactions that were performed in inert conditions were freshly distilled from sodium and degassed prior to use. All NMR spectra for these experiments were recorded at 293 K. For ^1^H-NMR a 400 MHz Avance 400 (Bruker, Karlsruhe, Germany) or a 300 MHz Advance 300 machine (Bruker, Karlsruhe, Germany) was used. These spectra were referenced internally to residual solvent resonance of CDCl_3_ (δ = 7.26 ppm) or DMSO (2.50). For ^13^C{^1^H}-NMR a Bruker Avance 400 (101 MHz) or Bruker Avance 500 (126 MHz) machine was used. These spectra were referenced internally to residual solvent resonance of CDCl_3_ (δ = 77.2 ppm) or DMSO-*d*_6_ (39.5). UV-vis spectra were recorded on a Hewlett Packard 8453 spectrophotometer (Waldbronn, Germany).

### 3.2. Synthetic Details

Compounds **1** [1] and **7** [2] were prepared according to literature reports and their analytical data matched those reported.

#### 3.2.1. Synthesis of 2-Azido-5-nitrophenol (**5**)

2-Amino-5-nitrophenol (72.4 mmol) was added to aq.HCl (6 M, 100 mL) at 0 °C. To this a solution of NaNO_2_ (96.5 mmol) in water (20 mL) was added dropwise. After stirring this mixture for 5 min sodium azide (96.5 mmol) that was predissolved in water (60 mL) was added dropwise and stirred for 45 min. The precipitate was extracted with chloroform and then washed with water. The organic layer was dried over MgSO_4_. On evaporation of the solvent a dark pink brownish solid was obtained (80% crude yield). This was purified further by flash chromatography over silica (EtOAc-Hex = 1:1) to give a dark pink solid (75% yield). ^1^H-NMR (400 MHz, chloroform-*d*) δ 7.87 (dd, *J* = 8.7, 2.5 Hz, 1H), 7.80 (d, *J* = 2.5 Hz, 1H), 7.19 (d, *J* = 8.7 Hz, 1H), 5.65 (s, 1H). ^13^C-NMR (75 MHz, chloroform-*d*) δ 147.46, 145.53, 133.08, 118.25, 116.92, 111.66.

#### 3.2.2. Synthesis of the Catalyst **CoP2**

Perfluorinated tetraphenylporphyrin TPPH_2_F_20_ (0.5 mmol, 480 mg) was refluxed in acetic acid (300 mL) with an excess of CoCl_2_·6H_2_O (2.6 mmol, 620 mg) and sodium acetate (15 mmol, 1.24 g) for 1 h. The mixture was allowed to cool down and the solvent was evaporated until most solids precipitated out. The red solids thus obtained were washed with water, aqueous sodium bicarbonate and finally with a minimum amount of cold methanol. The solid was then dried *in-vacuo* to give **CoP2** (90% yield). UV-vis spectrum (THF), λ_max_/nm: 408, 520, 558.

#### 3.2.3. Catalytic Reaction of Compound **1** to Give Product **3**

In a flame dried Schlenk flask loaded with a stirrer 1 mmol of **1** was added followed by 0.05 mmol of **CoP1**. The Schlenk flask was then evacuated and refilled with nitrogen (three times). Subsequently 4 mL of dry chlorobenzene was added, and the flask was thermostatted at 50 °C for 18 h. After evaporating the solvent the mixture was directly loaded on a silica gel column. The product was eluted with Hex:EtOAc (1:1) to give product **3** in 80% isolated yield. ^1^H-NMR (400 MHz, Chloroform-*d*) δ 7.79–7.72 (m, 1H), 7.45 (ddd, *J* = 8.3, 6.9, 1.6 Hz, 1H), 7.42–7.32 (m, 2H), 6.48 (s, 1H), 6.42 (s, 1H), 5.11 (s, 2H). ^13^C-NMR (75 MHz, CDCl_3_) δ 180.00, 149.72, 142.25, 133.23, 129.59, 128.29, 125.05, 115.56, 114.24, 103.61.

For the other reactions of compound **1** with other substrates, the same stoichiometry was used (also see the footnote in Table 1).

#### 3.2.4. Catalytic Reaction of Compound **5** to Give Product **6**

In a flame dried Schlenk flask loaded with a stirrer 1 mmol of **5** was added followed by 0.05 mmol of **CoP2**. The Schlenk flask was then evacuated and refilled with nitrogen (three times). Subsequently 4 mL of chlorobenzene was added, and the reaction was thermostatted at 50 °C for 18 h. After evaporating the solvent, the mixture was directly loaded on a silica gel column. The product was eluted with Hex:EtOAc (1:1) to give product **6** in 80% yield. ^1^H-NMR (300 MHz, Chloroform-*d*) δ 7.82–7.65 (m, 2H), 6.53 (d, *J* = 8.7 Hz, 1H), 5.27 (t, *J* = 2.4 Hz, 1H), 4.57 (s, 1H), 3.85 (dt, *J* = 9.7, 6.7 Hz, 1H), 3.62 (dt, *J* = 9.7, 6.6 Hz, 1H), 3.58–3.46 (m, 1H), 3.51–3.38 (m, 1H), 1.54 (dq, *J* = 8.6, 6.8 Hz, 2H), 1.29 (dt, *J* = 14.9, 7.4 Hz, 3H), 0.86 (t, *J* = 7.4 Hz, 4H).^13^C-NMR (75 MHz, CDCl_3_) δ 140.14, 139.16, 138.81, 119.53, 113.78, 112.78, 93.85, 68.60, 44.42, 31.54, 19.25, 13.86.

For the other reactions of compound **5** with other substrates, the same stoichiometry was used (also see footnote in Table 2).

#### 3.2.5. Catalytic Reaction of **7** to Give Compound **8**

In a flame dried Schlenk flask loaded with a stirrer compound **7** (67 mg, 0.5 mg) and **CoP1** (17 mg, 0.025 mmol) was added and the flask was evacuated and backfilled with dinitrogen (three times). Subsequently 4 mL of PhCl was added, and the reaction mixture was thermostatted at 50 °C for 18 h. The reaction mixture was then subjected to preparative TLC and a bright orange band was obtained which was analyzed and found to be compound **8**.

#### 3.2.6. Catalytic Reaction of Aminophenyl Azide **7** to Give **8**

In a flame dried Schlenk flask loaded with a stirrer compound **7** (67 mg, 0.5 mg) and **CoP1** (17 mg, 0.025 mmol) were added and the flask was evacuated and backfilled with dinitrogen (three times). Subsequently 4 mL of PhCl was added. The reaction mixture was then thermostatted at 50 °C for 18 h. The resulting mixture was subjected to preparative TLC and a bright orange band was obtained which was analyzed and found to be compound **8**. ^1^H-NMR (400 MHz, Chloroform-*d*) δ 7.68 (dd, *J* = 8.0, 1.6 Hz, 1H), 7.23–7.06 (m, 1H), 6.93–6.63 (m, 2H), 5.48 (s, 2H).^13^C-NMR (126 MHz, CDCl_3_) δ 143.11, 137.73, 131.37, 124.29, 117.66, 117.04.

### 3.3. Computational Details

Geometry optimizations were carried out with the Turbomole program package [44] coupled to the PQS Baker optimizer [45,46] via the BOpt package [47] at the DFT level using the b3-lyp [48,49,50] functional and def(2)-TZVP basis set [51] for the geometry optimizations of all stationary points. Grimme’s D3 dispersion corrections were used in all calculations (disp3, zero damping) [52]. All minima (no imaginary frequencies) and transition states (one imaginary frequency) were characterized by numerically calculating the Hessian matrix. ZPE and gas-phase thermal corrections (entropy and enthalpy, 298 K, 1 bar) from these analyses were calculated.

## 4. Conclusions

**Scheme 9:**
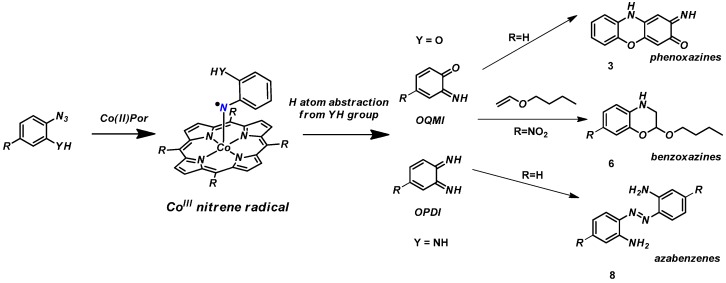
Transformations observed in this study.

While trying to employ *ortho*-YH substituted phenyl azides (Y = OH, NH_2_) as substrates in nitrene transfer reactions catalyzed by cobalt(II) porphyrins, we discovered that the hydrogen atom of the YH moiety is readily abstracted by the reactive cobalt(III) nitrene radical intermediate. This leads to formation of reactive intermediates like *o*-quinone monoimines (OQMI; Y = OH) and orthophenylinendiimines (OPDI; Y = NH_2_). These reactive compounds rapidly undergo several follow-up reactions (Scheme 9), thus preventing any direct (radical-type) coupling reactions of the nitrene radical intermediate with C=C or C≡C bonds of other substrates. The *o*-quinone monoimines (Y = OH) easily dimerize and produce phenoxizinone **3** under aerobic conditions. In the presence of 1-butoxyethene the *o*-quinone monoimine can also be trapped in an IEDDA reaction, producing benzoxazine **6**. Formation of orthophenylinendiimine (OPDI) from ortho-NH_2_-phenylazide (Y = NH_2_) is also associated with hydrogen atom abstraction of the Co(III) nitrene radical from the NH_2_ substituent in the ortho position. As a result, aza compound **8** is obtained. Attempts to react *ortho* substituted azides with other reaction partners by altering the reaction conditions were not successful. DFT computations are in agreement with the experiments; HAT from the ortho-YH substitutent (Y= O or NH) to the nitrene moiety has a (very) low barrier in both cases.

The observed facile HAT from the ortho-YH substitutent (Y= O or NH) to the nitrene moiety is perhaps a potential disadvantage of using these systems, as it prevents the initially anticipated radical-type coupling of the cobalt(III) nitrene radical intermediate to C=C and C≡C bonds of other substrates. On the other hand, it does provide a rather mild route to prepare highly reactive *o*-iminoquinonoids and o-phenylenediimines which can be employed in several follow up reactions. At the same time, the possibility of further reactivity of these intermediates occurring *in the coordination sphere of the catalyst* cannot be ruled out at the moment and might even be plausible based on the bond distance data that we obtained from the DFT optimized structures of these compounds after HAT. This suggests that diastereo- or enantioselective reactions may be accessible in synthetically useful transformations [42], mediated by chiral cobalt catalyst. This is a current topic of investigations in our group.

## Figures and Tables

**Table 1:** Reaction of *o*-hydroxy phenyl azide **1** with different substrates under reaction conditions ^a,b,^*.

Entry	Substrate	Expected Product(s)	Obtained Product
1	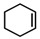	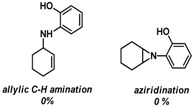	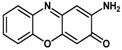
2	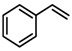	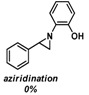	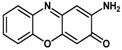
3	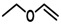	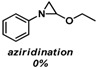	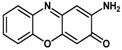

* *Reaction conditions*: ^a^: 0.5 mmol of azide **1**, 1 mmol of substrate, 5 mol% **CoP1** (w.r.t. azide), 4 mL toluene, 50 °C, 18 h; ^b^: 0.6 mmol of azide **1**, 15 mL of substrate, 5 mol% **CoP1**, 50 °C, 18 h. These different reaction conditions (^a,b^) gave identical results. No nitrene transfer to the solvent (toluene) was observed in any case under the applied reaction conditions.

**Table 2:** Reaction of azide **5** with different substrates ^a,b,*^.

Entry	Substrate	Expected Product(s)	Obtained Product
1	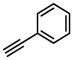	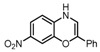	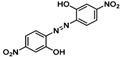 (~10%)
2	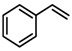	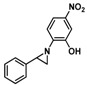	---
3	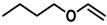	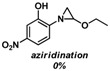	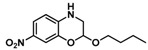 (80%)
4	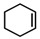	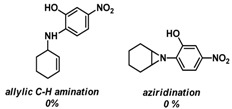	---

* Reaction conditions: ^a^ 0.5 mmol of azide **1**, 1 mmol of substrate, 5 mol% **CoP2** (w.r.t. azide), 4 mL toluene, 50 °C, 18 h. ^b^ 0.6 mmol of azide **1**, 15 mL of substrate, 5 mol% **CoP2**, 50 °C, 18 h. These different reaction conditions (^a,b^) gave identical results. No nitrene transfer to the solvent (toluene) was observed in any case under the applied reaction conditions.

**Table 3:** Changes of the N, O and Co atom spin populations during the HAT process shown in Figure 2.

Atom	2A	2B	2C
Co	11.1%	8.5%	60.6%
N	39.6%	33.5%	16.1%
O	5.3%	10.0%	9.4%

**Table 4:** Relevant bond length (Å) changes during the HAT process shown in Figure 2.

Structure	Co–N	N–C	C–C	C–O	N–H
2A	1.81769	1.32018	1.47138	1.34208	1.86203
2B	1.81731	1.31667	1.48868	1.30627	1.33591
2C	1.92289	1.32157	1.50424	1.24441	1.03461

**Table 5:** Changes of the N, O and Co atom spin populations during the HAT process shown in Figure 4.

Atom	3A	3B	3C
Co	8%	11%	63%
N	36%	24%	10%
O	10%	20%	9%

**Table 6:** Relevant bond length (Å) changes during the HAT process shown in Figure 4.

Structure	Co–N	N–C	C–C	C–N	N–H
3A	1.8411	1.3161	1.4835	1.3576	2.1848
3B	1.8521	1.3126	1.5060	1.3262	1.3213
3C	1.9604	1.3152	1.5022	1.4378	1.0340

## Supplementary Materials

Details of the newly synthesized compounds and catalytic reactions are available online at http://www.mdpi.com/1420-3049/21/2/242/s1. See the Supporting Information (SI) for NMR spectra and DFT tables.
