# Supplementary material for: Porphyrin Cobalt(III) “Nitrene Radical” Reactivity; Hydrogen Atom Transfer from Ortho-YH Substituents to the Nitrene Moiety of Cobalt-Bound Aryl Nitrene Intermediates (Y = O, NH)
Source: Molecules. 2016 Feb 20;21(2):242. doi: 10.3390/molecules21020242 (PMC6274064; doi:10.3390/molecules21020242)
Supplement: Supplementary file 1 [file molecules-21-00242-s001.pdf]

Supplementary Materials: Porphyrin Cobalt(III) "Nitrene Radical" Reactivity; Hydrogen Atom Transfer from Ortho-YH Substituents to the Nitrene Moiety of Cobalt-Bound Aryl Nitrene Intermediates (Y = O, NH)

Monalisa Goswami, Christophe Rebreyend and Bas de Bruin

1. Experimental Section

All NMR spectra were recorded at 293 K.

^{1}H-NMR: Bruker Avance 400 (400 MHz) or Mercury 300 (300 MHz), referenced internally to residual solvent resonance of $CDCl_3$ (δ = 7.26 ppm) or DMSO (2.50).

^{13}C{^{1}H}-NMR: Bruker Avance 400 (101 MHz) or Bruker Avance 500 (126 MHz), referenced internally to residual solvent resonance of $CDCl_3$ (δ = 77.2 ppm) or DMSO-d_6 (39.5).

2. Spectra

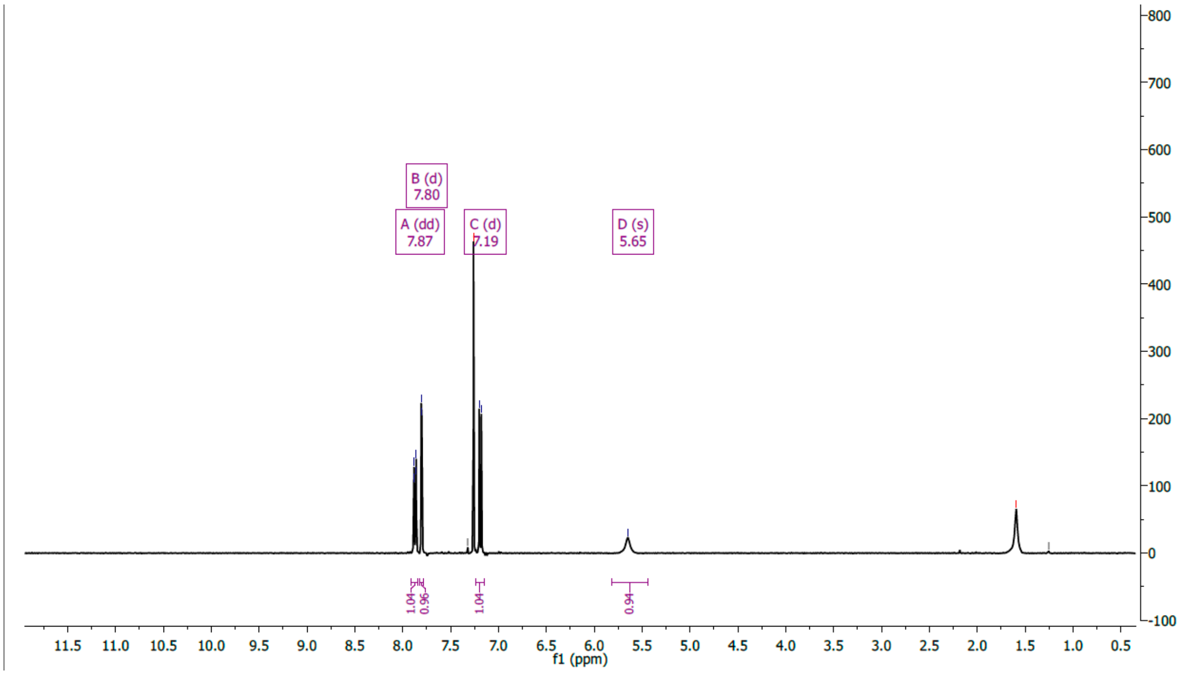

Figure S1. *Cont.*

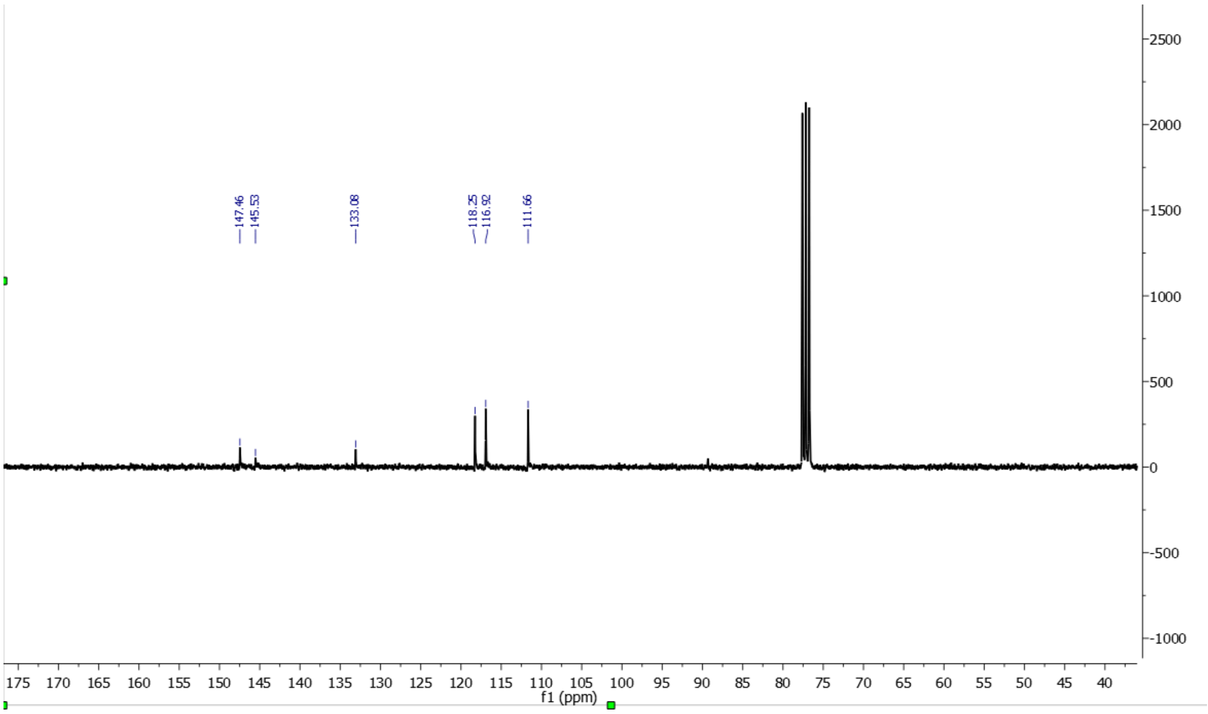

Figure S1. NMR spectra of compound 2-azido-5-nitrophenol **5**.

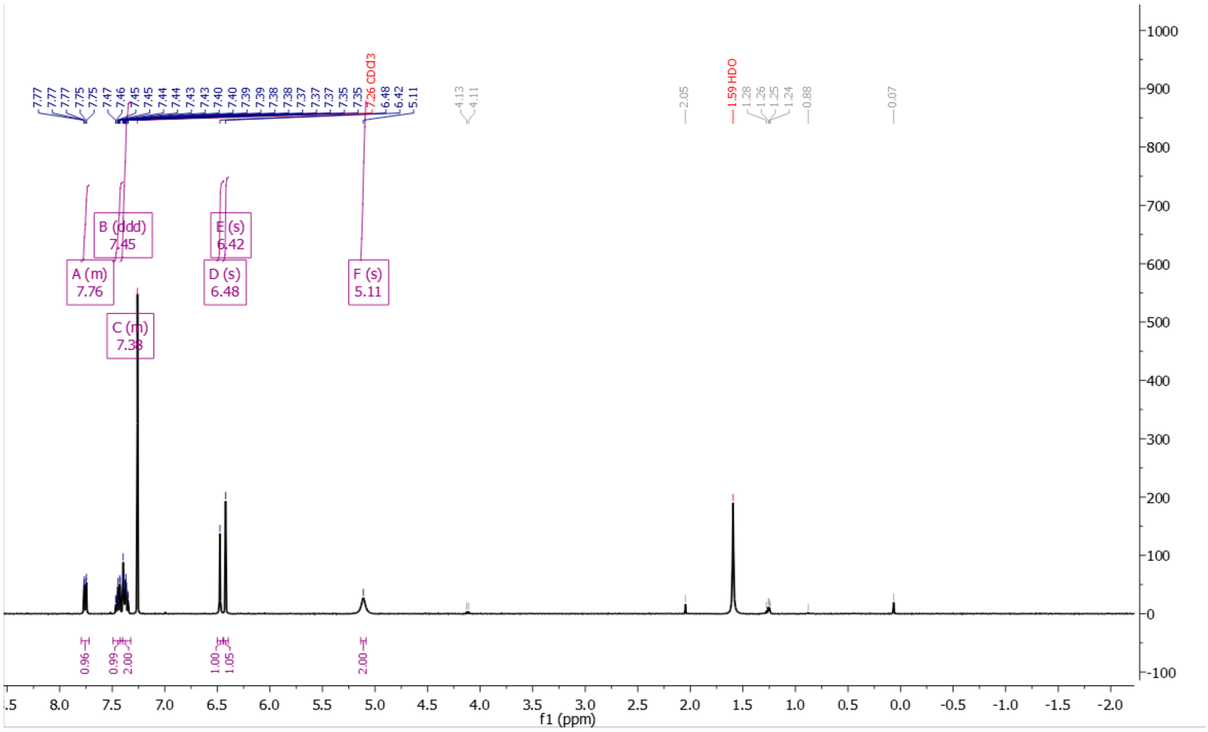

Figure S2. *Cont.*

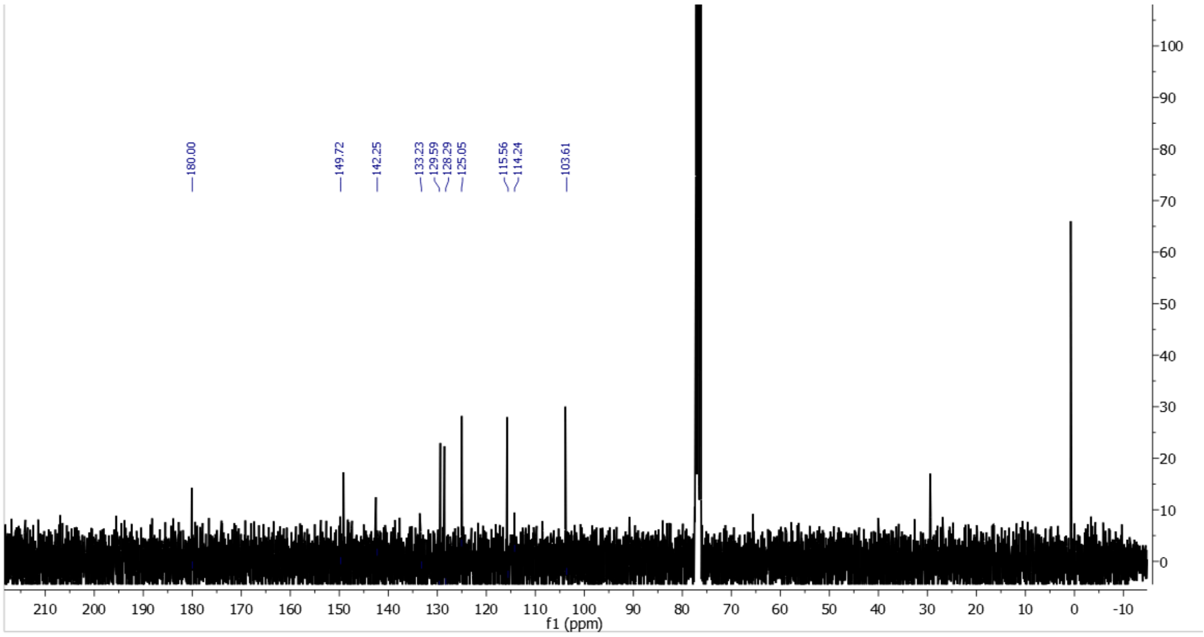

Figure S2. NMR spectra of product **3**.

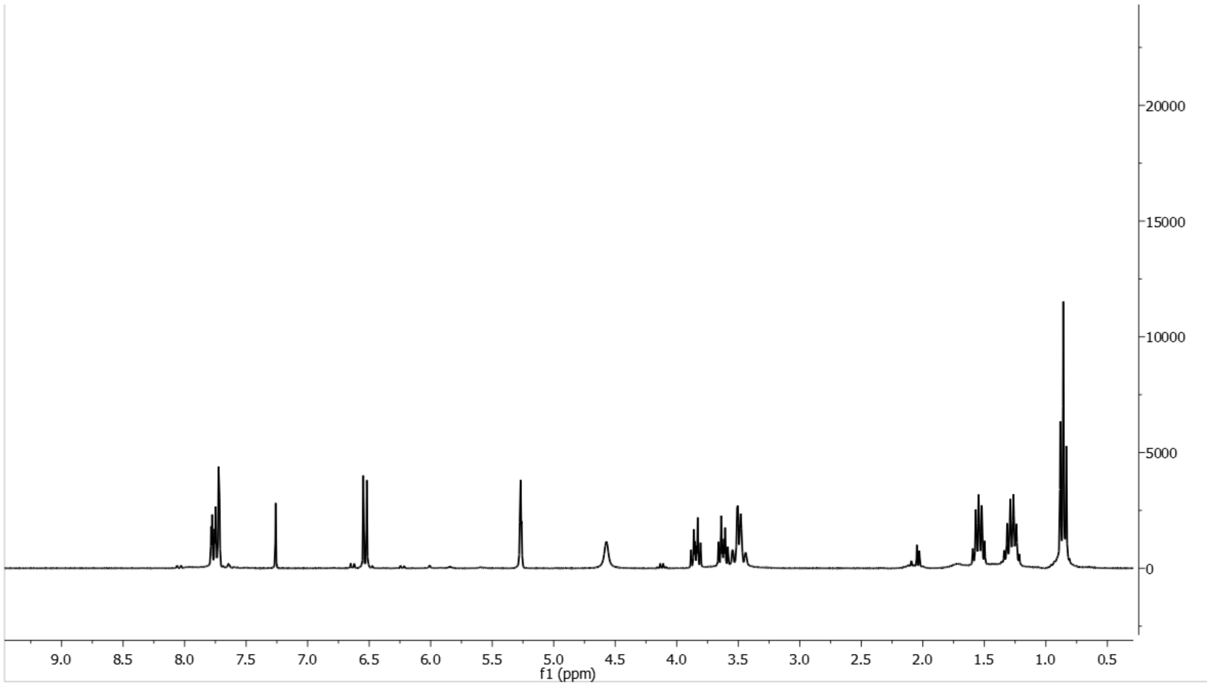

Figure S3. *Cont.*

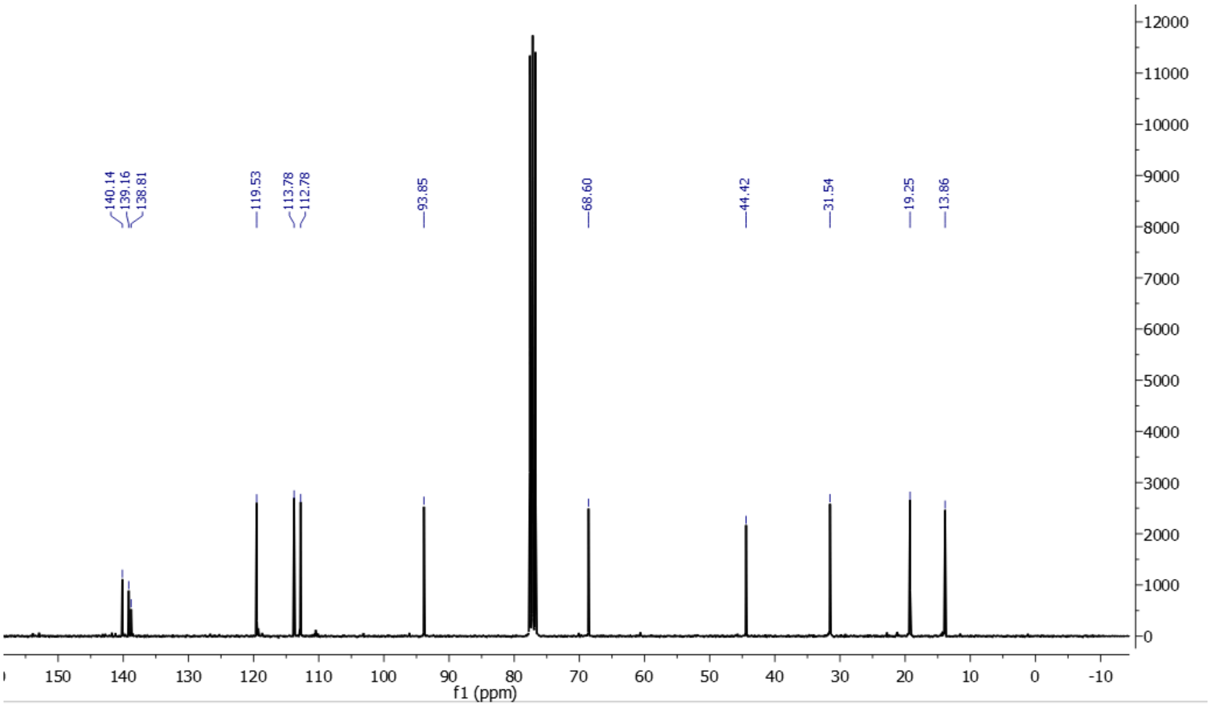

Figure S3. NMR spectra of product **6**.

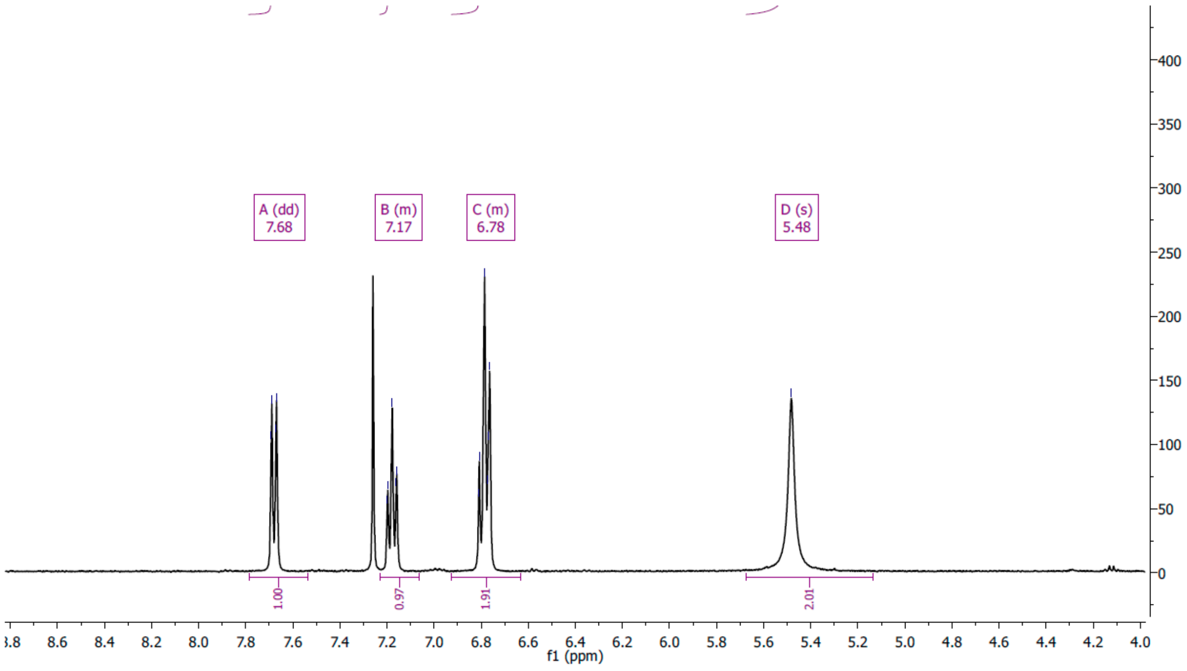

Figure S4. *Cont.*

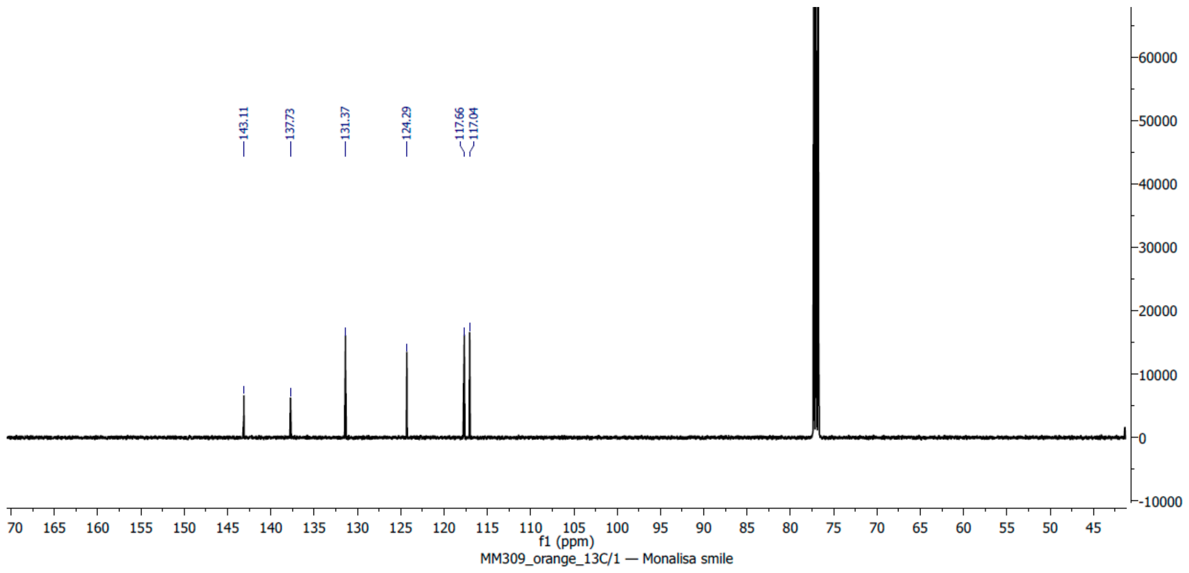

Figure S4. NMR spectra of product **8**.

3. Computational Tables

Table S1. SCF energies, enthalpies and free energies of the compounds involved in the HAT step described in Figure 2 in the main text in Hartree (BP86, def2-TZVP, disp3).

	E (SCF)	G	G_corr	H_corr	H
**2A**	−2938.29618	−2937.99037	0.30581	0.39329	−2937.90289
**2B**	−2938.28968	−2937.98881	0.30087	0.38904	−2937.90064
**2C**	−2938.31264	−2938.0076	0.30504	0.39402	−2937.91862

Table S2. SCF energies, enthalpies and free energies of the compounds involved in the HAT step described in Figure 4 in the main text in Hartree (BP86, def2-TZVP, disp3).

	E (SCF)	G	G_corr	H_corr	H
**3A**	−2918.49820	−2918.18056	0.31763	0.40610	−2918.09210
**3B**	−2918.47866	−2918.16598	0.31268	0.40124	−2918.07742
**3C**	−2918.50309	−2918.18553	0.31755	0.40644	−2918.09665

Table S3. SCF energies, enthalpies and free energies of the compounds involved in the HAT step described in Figure 6 in the main text in Hartree (BP86, def2-TZVP, disp3).

	E (SCF)	G	G_corr	H_corr	H
**4A**	−3227.02126	−3226.62744	0.38199	0.5198	−3226.50146
**4B**	−3227.02225	−3226.60617	0.41609	0.52110	−3226.97014
**4C**	−3227.06315	−3226.64472	0.41843	0.52372	−3277.58687

3.1. X,Y,Z Coordinates of Compound 2A in Figure 2 in Main Text (BP86, def2-TZVP, disp3)

52
Energy = −2938.3767558710

C	−0.1000685	0.0070748	2.6874809
C	0.5318198	0.0973631	3.9773508
C	1.8382627	0.4243430	3.7570128
C	1.9930823	0.5947604	2.3362556

H	0.0300678	−0.0911988	4.9222908
N	0.8026184	0.3229538	1.6848677
C	3.1264653	1.1123197	1.7304336
H	4.0088926	1.2770414	2.3478306
C	3.1533687	1.5563900	0.4183848
C	2.5370051	2.0512071	−1.6316560
C	3.8333686	2.6497223	−1.4289484
C	4.2284188	2.3170485	−0.1666014
H	4.3675806	3.2284919	−2.1773163
H	5.1508141	2.5751033	0.3463224
N	2.1161533	1.4146612	−0.4822334
C	1.8734009	2.0246507	−2.8480705
H	2.3170602	2.5665626	−3.6829197
C	0.7456916	1.2591285	−3.0986344
C	−0.8653401	−0.2171744	−2.8704433
C	−0.7912032	0.1035675	−4.2733262
C	0.1885080	1.0423422	−4.4111031
H	−1.4322234	−0.3255861	−5.0383733
H	0.5311694	1.5385448	−5.3148578
N	0.0695307	0.5082744	−2.1590206
C	−1.8071440	−1.0611882	−2.3050390
H	−2.4932077	−1.5858504	−2.9692861
C	−1.3914286	−0.4482343	2.4763084
H	−2.0091901	−0.6643142	3.3472871
C	−1.8879223	−0.7770607	1.2251548
C	−3.1108074	−1.5015395	0.9994232
C	−3.1685314	−1.7759201	−0.3361843
C	−2.0122119	−1.1668951	−0.9388642
H	−3.8143156	−1.7779853	1.7797068
H	−3.9394704	−2.3093582	−0.8851543
N	−1.2228654	−0.5680053	0.0275789
H	2.6298477	0.5791539	4.4849028
Co	0.5238868	0.2631578	−0.2487425
N	1.5069792	−1.2158937	−0.6359598
C	1.5402940	−2.3863351	−0.0261767
C	1.9939175	−5.0055626	0.9094028
C	2.5186268	−3.2823381	−0.6625876
C	0.8184004	−2.9077756	1.0962364
C	1.0403584	−4.1871565	1.5515880
C	2.7349192	−4.5675039	−0.1930995
H	0.0869390	−2.2738288	1.5841691
H	0.4953531	−4.5895653	2.4030500
H	3.4645650	−5.2207404	−0.6673177
O	3.1928934	−2.7925309	−1.7145499
H	2.8147426	−1.8643719	−1.7919898
N	2.2184746	−6.3611540	1.4149137
O	3.0616254	−7.0649812	0.8375141
O	1.5527328	−6.7330572	2.3956437

3.2. X,Y,Z Coordinates of Compound 2B in Figure 2 in Main Text (BP86, def2-TZVP, disp3)

52

Energy = −2938.3703671230

C	−0.1099678	0.0354651	2.6910147
C	0.5250115	0.1219400	3.9795563
C	1.8319685	0.4435863	3.7566796
C	1.9844156	0.6117139	2.3356431
H	0.0242290	−0.0657501	4.9251323
N	0.7900342	0.3505792	1.6864044
C	3.1242809	1.1079354	1.7257807
H	4.0080664	1.2683494	2.3423602
C	3.1630427	1.5276146	0.4068251
C	2.5667162	1.9959036	−1.6561419
C	3.8695748	2.5779797	−1.4550127
C	4.2517323	2.2615475	−0.1848443
H	4.4178611	3.1334225	−2.2107017
H	5.1754056	2.5131907	0.3287304
N	2.1271709	1.3851443	−0.4974901
C	1.9102954	1.9593772	−2.8751251
H	2.3673865	2.4783494	−3.7171235
C	0.7728729	1.2069666	−3.1163606
C	−0.8677199	−0.2337719	−2.8663595
C	−0.7809653	0.0582967	−4.2739862
C	0.2171819	0.9749275	−4.4256004
H	−1.4264081	−0.3741738	−5.0333201
H	0.5744283	1.4453249	−5.3373715
N	0.0792813	0.4868705	−2.1631497
C	−1.8312830	−1.0451180	−2.2914616
H	−2.5235377	−1.5676423	−2.9508640
C	−1.4051276	−0.4096000	2.4848041
H	−2.0209821	−0.6214538	3.3581439
C	−1.9102050	−0.7306887	1.2358069
C	−3.1420882	−1.4412347	1.0155958
C	−3.2065671	−1.7188524	−0.3187129
C	−2.0436320	−1.1272067	−0.9254323
H	−3.8460991	−1.7076628	1.7989044
H	−3.9841110	−2.2460804	−0.8642899
N	−1.2472163	−0.5288750	0.0358450
H	2.6265832	0.5927955	4.4823000
Co	0.5119913	0.2816263	−0.2495060
N	1.4600569	−1.2347963	−0.5723789
C	1.4915754	−2.4005717	0.0388386
C	2.0589261	−5.0134006	0.8464985
C	2.5437612	−3.2061406	−0.6394905
C	0.7664801	−2.9781150	1.1197913
C	1.0506164	−4.2668094	1.5105797
C	2.8098994	−4.5111827	−0.2163025
H	−0.0033442	−2.4025040	1.6249388
H	0.5157299	−4.7417875	2.3302028
H	3.5769650	−5.1168667	−0.6937694
O	3.1292702	−2.5551174	−1.6088707
H	2.4403934	−1.5514817	−1.4228539
N	2.3233250	−6.3819665	1.3063974
O	3.2064181	−7.0302968	0.7256929

O	1.6483090	−6.8187621	2.2540530

3.3. X,Y,Z Coordinates of Compound 2C in Figure 2 in Main Text (BP86, def2-TZVP, disp3)

52
Energy = −2938.3948562410

C	−1.0180320	−0.2089291	2.8325966
C	−0.6492225	−0.1190427	4.2219149
C	0.6359665	0.3376159	4.2610607
C	1.0682098	0.4848884	2.8950045
H	−1.3122067	−0.3496910	5.0511710
N	0.0447237	0.1532515	2.0253124
C	2.3565449	0.8254137	2.5132350
H	3.0762389	1.0638864	3.2958283
C	2.8113415	0.7712015	1.2056690
C	2.9142001	0.3512325	−0.9503790
C	4.2641857	0.5760582	−0.5042191
C	4.1967160	0.8706630	0.8264410
H	5.1394309	0.5376864	−1.1465801
H	5.0067737	1.1080413	1.5103270
N	2.0289276	0.4729559	0.1042141
C	2.5516891	0.1383907	−2.2714646
H	3.3456265	0.0372627	−3.0105330
C	1.2424177	0.1658504	−2.7222706
C	−0.9492001	0.3488280	−2.7715841
C	−0.4994480	0.3304418	−4.1403504
C	0.8561156	0.1853480	−4.1091585
H	−1.1554864	0.3928742	−5.0039599
H	1.5503232	0.1158455	−4.9417160
N	0.1283125	0.2775917	−1.9098776
C	−2.2799617	0.3254850	−2.3852961
H	−3.0384693	0.3927493	−3.1646825
C	−2.2950383	−0.5010153	2.3811170
H	−3.0477637	−0.7745471	3.1200986
C	−2.7017497	−0.3497084	1.0649663
C	−4.0719613	−0.4234395	0.6237789
C	−4.0766116	−0.1103489	−0.7033383
C	−2.7041964	0.1139051	−1.0832361
H	−4.9139130	−0.6623937	1.2675482
H	−4.9212034	−0.0502415	−1.3840779
N	−1.8722306	−0.0139016	0.0129203
H	1.2550016	0.5490587	5.1285668
Co	0.1012362	0.0600361	0.0560039
N	0.3071564	−1.8290015	−0.2383752
C	1.4090976	−2.5555964	−0.1725827
C	3.7636866	−4.0712015	−0.1765730
C	1.6845726	−3.4914502	−1.3175816
C	2.3279814	−2.4695478	0.9198710
C	3.4804224	−3.2016956	0.9250129
C	2.9299086	−4.2372240	−1.2463925
H	2.0830818	−1.8032449	1.7453041
H	4.1930276	−3.1472249	1.7443099

H	3.1859470	−4.9063467	−2.0654386
O	0.8886326	−3.5855275	−2.2695200
N	5.0480029	−4.8245399	−0.1506664
O	5.2730143	−5.6192022	−1.0668237
O	5.8171148	−4.6014789	0.7924751
H	−0.2100509	−2.1031656	−1.0914606

3.4. X,Y,Z Coordinates of Compound 3A in Figure 4 in Main Text (BP86, def2-TZVP, disp3)

53

Energy = −2918.4981954630

C	−0.1542683	0.0308018	2.6479195
C	0.4717602	0.1422034	3.9392296
C	1.7725703	0.4912208	3.7200999
C	1.9286694	0.6524414	2.2980215
H	−0.0296275	−0.0475954	4.8841421
N	0.7459307	0.3527066	1.6451340
C	3.0546469	1.1868130	1.6927269
H	3.9304786	1.3741409	2.3132987
C	3.0790851	1.6218678	0.3772752
C	2.4621235	2.0896748	−1.6779938
C	3.7479820	2.7119249	−1.4761631
C	4.1436062	2.3969283	−0.2091656
H	4.2737708	3.2963256	−2.2263160
H	5.0580492	2.6777669	0.3063266
N	2.0491954	1.4538923	−0.5259690
C	1.8004475	2.0470699	−2.8955301
H	2.2375143	2.5918642	−3.7321771
C	0.6795042	1.2702369	−3.1438219
C	−0.9207448	−0.2157051	−2.9113155
C	−0.8518756	0.1036606	−4.3152295
C	0.1222514	1.0480652	−4.4559244
H	−1.4928069	−0.3293422	−5.0783390
H	0.4590273	1.5467308	−5.3606804
N	0.0106283	0.5156003	−2.2028832
C	−1.8548254	−1.0668594	−2.3429599
H	−2.5401149	−1.5953907	−3.0051449
C	−1.4385959	−0.4445499	2.4371261
H	−2.0543989	−0.6645288	3.3085705
C	−1.9312017	−0.7833636	1.1869331
C	−3.1492312	−1.5173537	0.9634720
C	−3.2069824	−1.7919566	−0.3719345
C	−2.0551427	−1.1748592	−0.9761532
H	−3.8497516	−1.7983879	1.7448435
H	−3.9753564	−2.3301094	−0.9201165
N	−1.2679857	−0.5716124	−0.0109132
H	2.5587986	0.6671956	4.4490795
Co	0.4689096	0.2787055	−0.2876759
N	1.4821356	−1.1919847	−0.6847613
C	1.5347026	−2.3598829	−0.0834682
C	1.9936769	−4.9506639	0.9684598
C	2.5123723	−3.2860811	−0.7008099

C	0.8150696	−2.8554123	1.0590505
C	1.0342058	−4.1087118	1.5735772
C	2.7215165	−4.5518794	−0.1516551
H	0.0832583	−2.2019148	1.5179310
H	0.4881889	−4.4723406	2.4416441
H	3.4461501	−5.2400776	−0.5864517
N	2.2322200	−6.2763981	1.5275140
O	3.0879261	−6.9962235	0.9827435
O	1.5677478	−6.6187757	2.5214032
N	3.1636012	−2.8512293	−1.8106767
H	3.0129225	−1.8754551	−2.0609494
H	3.9569820	−3.3584901	−2.1791670

3.5. X,Y,Z Coordinates of Compound 3B in Figure 4 in Main Text (BP86, def2-TZVP, disp3)

53
Energy = −2918.4786616970

C	−0.1593583	−0.0014974	2.6180856
C	0.4873922	0.0682778	3.9023353
C	1.7916415	0.3935517	3.6714716
C	1.9307839	0.5765904	2.2504100
H	−0.0049132	−0.1308775	4.8500713
N	0.7307161	0.3240292	1.6088086
C	3.0689345	1.0714778	1.6366906
H	3.9560100	1.2265520	2.2499905
C	3.1055733	1.4917941	0.3177453
C	2.5075539	1.9622970	−1.7438062
C	3.8177583	2.5301945	−1.5485727
C	4.1998834	2.2141585	−0.2780758
H	4.3682773	3.0802949	−2.3066597
H	5.1263685	2.4598888	0.2336021
N	2.0658714	1.3567992	−0.5833311
C	1.8422566	1.9407833	−2.9587186
H	2.3018136	2.4566366	−3.8014391
C	0.6855109	1.2165223	−3.1942366
C	−0.9929466	−0.1764113	−2.9369651
C	−0.9184767	0.1301289	−4.3427352
C	0.1056929	1.0167041	−4.4987346
H	−1.5895056	−0.2714472	−5.0968796
H	0.4629046	1.4886783	−5.4098252
N	−0.0139039	0.5043029	−2.2396772
C	−1.9722647	−0.9667921	−2.3589830
H	−2.6906997	−1.4565467	−3.0155918
C	−1.4602983	−0.4332404	2.4212155
H	−2.0667088	−0.6532611	3.2992151
C	−1.9885030	−0.7221086	1.1740559
C	−3.2401153	−1.4002488	0.9599407
C	−3.3334991	−1.6446538	−0.3789281
C	−2.1655530	−1.0678591	−0.9914416
H	−3.9368501	−1.6681545	1.7493092
H	−4.1322916	−2.1406153	−0.9231611
N	−1.3387995	−0.5110396	−0.0313733

H	2.5932286	0.5347245	4.3911183
Co	0.4333487	0.2765846	−0.3265012
N	1.3868922	−1.2552198	−0.6625962
C	1.4819520	−2.3905188	−0.0137602
C	2.1417536	−4.9379927	0.9806430
C	2.6570496	−3.1581497	−0.5507523
C	0.6989178	−2.9854252	1.0204615
C	1.0209152	−4.2312669	1.5008268
C	2.9583998	−4.4281108	−0.0198573
H	−0.1543452	−2.4440978	1.4180005
H	0.4346101	−4.7060125	2.2845342
H	3.8150571	−5.0037131	−0.3673338
N	2.4510066	−6.2567178	1.5345177
O	3.4233018	−6.8736659	1.0686535
O	1.7248500	−6.6928615	2.4452308
N	3.2545103	−2.4514799	−1.5018937
H	2.4540113	−1.4322874	−1.4198729
H	4.0872848	−2.8086983	−1.9622304

3.6. X,Y,Z Coordinates of Compound 3C in Figure 4 in main text (BP86, def2-TZVP, disp3)

53
Energy = −2918.5030858880

C	−0.2934206	−0.4024895	2.4797454
C	0.3928664	−0.5819766	3.7329208
C	1.7226607	−0.4062611	3.4829453
C	1.8431930	−0.0687636	2.0885846
H	−0.0930349	−0.8381824	4.6702613
N	0.6025312	−0.0668704	1.4800062
C	3.0332300	0.2812397	1.4699073
H	3.9512944	0.2146878	2.0527135
C	3.1099525	0.8168707	0.1950142
C	2.5498442	1.6083472	−1.7778578
C	3.9480438	1.9023154	−1.5913465
C	4.3017506	1.3854668	−0.3798503
H	4.5704050	2.4179349	−2.3173563
H	5.2728849	1.3998928	0.1069808
N	2.0392378	0.9661755	−0.6668506
C	1.8516486	1.8467664	−2.9507784
H	2.3675018	2.3727238	−3.7537771
C	0.5747702	1.3717727	−3.2022606
C	−1.3236202	0.2825848	−3.0146281
C	−1.2510862	0.7626767	−4.3720107
C	−0.0841010	1.4590518	−4.4818110
H	−2.0162840	0.5967163	−5.1252580
H	0.3192103	1.9766896	−5.3478109
N	−0.2037263	0.6666172	−2.3038176
C	−2.4091457	−0.3957276	−2.4839088
H	−3.2301009	−0.6486138	−3.1542572
C	−1.6379163	−0.6727310	2.2847368
H	−2.2373587	−0.9400178	3.1546304
C	−2.2422146	−0.7209807	1.0382219

C	−3.5892090	−1.1766464	0.8055983
C	−3.7802110	−1.1702415	−0.5450671
C	−2.5637108	−0.6734130	−1.1355458
H	−4.2832643	−1.4704719	1.5883096
H	−4.6687813	−1.4466266	−1.1059022
N	−1.6217945	−0.4109853	−0.1581825
H	2.5574291	−0.4713679	4.1751326
Co	0.2480800	0.1457254	−0.4523058
N	1.0712951	−1.5290537	−1.0151113
C	1.5800588	−2.4698098	−0.2415239
C	2.7451659	−4.3688221	1.4693475
C	2.9658475	−2.9566697	−0.5082344
C	0.8432389	−3.0180884	0.8586397
C	1.3993549	−3.9356801	1.7006142
C	3.5033820	−3.9270034	0.4237751
H	−0.1774315	−2.6684863	1.0052069
H	0.8525253	−4.3510966	2.5433355
H	4.5231047	−4.2914839	0.2990339
N	3.3428882	−5.3368663	2.4215519
O	4.4863691	−5.7464506	2.1958754
O	2.6563921	−5.6729147	3.3940192
N	3.5983048	−2.4541744	−1.5342682
H	1.7507437	−1.3209306	−1.7656125
H	4.5459222	−2.8436557	−1.6095985

3.7. X,Y,Z Coordinates of Compound 4B in Figure 6 in main text (BP86, def2-TZVP, disp3)

67
Energy = −3227.0221914460

C	0.0700493	−0.3051630	2.3628844
C	1.0502664	−0.4052898	3.4139151
C	2.2462227	−0.6589901	2.8131434
C	1.9985259	−0.6884479	1.3942441
H	0.8282018	−0.3091999	4.4730579
N	0.6620631	−0.4613594	1.1225707
C	2.9807805	−0.9055270	0.4449747
H	3.9946760	−1.0969017	0.7945658
C	2.7635897	−0.8537793	−0.9203220
C	1.8037393	−0.6362077	−2.8879019
C	3.2032818	−0.8669247	−3.1299471
C	3.7989692	−1.0038848	−1.9086669
H	3.6565987	−0.9024883	−4.1168292
H	4.8481737	−1.1695771	−1.6787237
N	1.5373149	−0.6369678	−1.5262437
C	0.8693894	−0.4140079	−3.8841549
H	1.2114334	−0.4184240	−4.9185027
C	−0.4768028	−0.2070760	−3.6475663
C	−2.4408964	−0.1313778	−2.6735783
C	−2.6667293	0.0761356	−4.0818473
C	−1.4448347	0.0445313	−4.6839600
H	−3.6428209	0.2391011	−4.5306081
H	−1.1984405	0.1557196	−5.7357961

N −1.0910622 −0.2792768 −2.4109660
C −3.4494599 −0.1474839 −1.7270755
H −4.4786456 −0.0751195 −2.0779287
C −1.2860796 −0.1566152 2.5927331
H −1.6205721 −0.0719363 3.6264562
C −2.2472730 −0.1414616 1.5970450
C −3.6648054 −0.0535151 1.8439529
C −4.2769503 −0.0869819 0.6263401
C −3.2304115 −0.1782562 −0.3609008
H −4.1143158 0.0278743 2.8299675
H −5.3374818 −0.0321980 0.3961537
N −1.9905396 −0.2135007 0.2424250
H 3.2200187 −0.8084164 3.2710206
Co −0.2394801 −0.6014220 −0.6476638
N −0.5817213 −2.4565176 −0.5987265
C 0.2203713 −3.2283814 0.2238724
C 1.8346186 −4.6144083 2.0321629
C 1.4545145 −3.8152948 −0.2164643
C −0.1841594 −3.4266804 1.5670438
C 0.6107766 −4.0962116 2.4795702
C 2.2554356 −4.4972308 0.7137229
H −1.1293966 −2.9790335 1.8729650
H 0.3203428 −4.2172355 3.5200874
H 3.2178120 −4.9165641 0.4224292
N 2.7212417 −5.2899954 2.9972882
O 3.8080618 −5.7202005 2.5866406
O 2.3347107 −5.3828663 4.1695221
N 1.8257513 −3.7128072 −1.5320226
H 1.3427354 −2.9912852 −2.0626751
H 2.8159857 −3.7799236 −1.7415046
C −0.9386092 −3.7212466 −3.0757589
H −2.0926977 −4.0805797 −1.2207595
C −1.3863235 −3.6555406 −1.9135040
C −0.3434070 −3.6463519 −4.3446257
C 0.8457827 −3.4412291 −6.9009004
C −1.0119159 −2.9915852 −5.4110545
C 0.9240444 −4.2297536 −4.6083546

3.8. X,Y,Z Coordinates of Compound 4C in Figure 6 in main text (BP86, def2-TZVP, disp3)

67
Energy = −3227.0631494640
C 0.6557974 −1.4131167 1.8356173
C 1.7543772 −1.7245754 2.7132858
C 2.8410438 −1.0580103 2.2321900
C 2.4093291 −0.3575083 1.0487403
H 1.6801109 −2.3632924 3.5887997
N 1.0663197 −0.5852116 0.8049367
C 3.2486443 0.4123381 0.2617320
H 4.2860730 0.5219369 0.5777456
C 2.8616974 1.0252159 −0.9171917
C 1.6746247 1.7007622 −2.6362584

C	3.0190242	2.1716187	−2.8484692
C	3.7525871	1.7653791	−1.7726900
H	3.3366871	2.7619201	−3.7035950
H	4.8029687	1.9444616	−1.5594623
N	1.5857110	0.9807449	−1.4577715
C	0.6076171	2.0128936	−3.4614088
H	0.8119694	2.5903362	−4.3627269
C	−0.7100104	1.6937973	−3.1834145
C	−2.5155063	0.8587141	−2.2534752
C	−2.9456975	1.5779928	−3.4246990
C	−1.8241377	2.0902228	−4.0063658
H	−3.9793447	1.6642292	−3.7481522
H	−1.7392892	2.6912124	−4.9075488
N	−1.1435153	0.9518537	−2.0971904
C	−3.3613776	0.1183377	−1.4468195
H	−4.4213942	0.1028154	−1.6989654
C	−0.6385650	−1.8556301	2.0334395
H	−0.8274969	−2.5193698	2.8763475
C	−1.7138735	−1.4954699	1.2398878
C	−3.0661164	−1.9310783	1.4744431
C	−3.8333944	−1.3801174	0.4907873
C	−2.9480110	−0.6095159	−0.3444119
H	−3.3710526	−2.5700485	2.2985177
H	−4.9051801	−1.4669139	0.3348455
N	−1.6478768	−0.6862430	0.1206046
H	3.8522465	−1.0312959	2.6289227
Co	−0.0220617	0.0197618	−0.7549610
N	0.1421707	−1.6470245	−1.8883148
C	0.4988680	−2.7949530	−1.1523626
C	1.3365972	−4.9476094	0.3712760
C	1.8914868	−3.0271402	−0.9734315
C	−0.4373486	−3.6778886	−0.5976745
C	−0.0340963	−4.7638518	0.1673516
C	2.2995185	−4.1086680	−0.1830174
H	−0.7472652	−5.4488151	0.6181425
H	3.3540121	−4.3117079	−0.0045192
N	1.7881854	−6.0724699	1.2153260
O	3.0055909	−6.2163343	1.3882202
O	0.9245013	−6.8082808	1.7091618
N	2.7879422	−2.2244654	−1.6479135
H	2.3860143	−1.3430306	−1.9685244
H	3.7072245	−2.1253960	−1.2302088
C	−0.9485032	−3.0263379	−3.5457392
C	−0.6173029	−4.3816720	−3.6358775
C	0.0852411	−7.1174543	−3.7024244
C	0.7449430	−4.7873365	−3.7760488
C	−1.6129430	−5.3961501	−3.5574238
C	−1.2594121	−6.7387854	−3.5856394
C	1.0801417	−6.1333704	−3.7983190
H	1.5179342	−4.0196648	−3.8251779
